# Peripheral Neuropathy in Diabetes Mellitus: Pathogenetic Mechanisms and Diagnostic Options

**DOI:** 10.3390/ijms24043554

**Published:** 2023-02-10

**Authors:** Raffaele Galiero, Alfredo Caturano, Erica Vetrano, Domenico Beccia, Chiara Brin, Maria Alfano, Jessica Di Salvo, Raffaella Epifani, Alessia Piacevole, Giuseppina Tagliaferri, Maria Rocco, Ilaria Iadicicco, Giovanni Docimo, Luca Rinaldi, Celestino Sardu, Teresa Salvatore, Raffaele Marfella, Ferdinando Carlo Sasso

**Affiliations:** 1 Department of Advanced Medical and Surgical Sciences, University of Campania “Luigi Vanvitelli”, I-80138 Naples, Italy; 2 Department of Precision Medicine, University of Campania “Luigi Vanvitelli”, I-80138 Naples, Italy

**Keywords:** type 2 diabetes mellitus, type 1 diabetes mellitus, diabetic peripheral neuropathy, pathophysiology, diagnosis

## Abstract

Diabetic neuropathy (DN) is one of the main microvascular complications of both type 1 and type 2 diabetes mellitus. Sometimes, this could already be present at the time of diagnosis for type 2 diabetes mellitus (T2DM), while it appears in subjects with type 1 diabetes mellitus (T1DM) almost 10 years after the onset of the disease. The impairment can involve both somatic fibers of the peripheral nervous system, with sensory-motor manifestations, as well as the autonomic system, with neurovegetative multiorgan manifestations through an impairment of sympathetic/parasympathetic conduction. It seems that, both indirectly and directly, the hyperglycemic state and oxygen delivery reduction through the vasa nervorum can determine inflammatory damage, which in turn is responsible for the alteration of the activity of the nerves. The symptoms and signs are therefore various, although symmetrical painful somatic neuropathy at the level of the lower limbs seems the most frequent manifestation. The pathophysiological aspects underlying the onset and progression of DN are not entirely clear. The purpose of this review is to shed light on the most recent discoveries in the pathophysiological and diagnostic fields concerning this complex and frequent complication of diabetes mellitus.

## 1. Introduction

Manifestations of diabetic neuropathy (DN) vary and depend on the nervous system involved (peripheral sensory/motor or vegetative), thus determining a complex picture of symptoms and signs depending on the organ involved. However, peripheral neuropathy is the most common and, as well described by recent guidelines, could affect almost 50% of individuals with diabetes mellitus (DM) during their lifetime [1].

Peripheral neuropathy (PN) is one of the main microvascular complications of both type 1 (T1DM) and type 2 diabetes mellitus (T2DM), more frequent than nephropathy and retinopathy, and is the leading cause of lower limb amputation in western countries [2,3,4].

However, there is a difference in the lifetime prevalence of peripheral neuropathy between T1DM and T2DM. As demonstrated by some trials, diabetic peripheral neuropathy (DPN) often is not present at the moment of diagnosis, while it could appear after at least 10 years of disease duration, and could affect up to 34% of subjects after ~25 years [5,6]. In contrast, DPN may already be present at the time of diagnosis in individuals with T2DM, and in this case, the prevalence increases together with the increasing age of the patient and the time duration of the diabetes [2,7,8]. In addition, some authors have observed that people with pre-diabetes could exhibit sensory neuropathy or neuropathic pain. The precise prevalence of pre-diabetic neuropathy is unknown, but it seems to be intermediate between individuals with normoglycemia and those with overt diabetes [9]. The estimated global prevalence of diabetes is almost 10% (500 million people) in 20–79-year-old subjects, and this prevalence will grow up to 11–12% (almost 700 million) in 2045 [10,11]. Based on these data, it is therefore clear that the prevalence of DN is also destined to increase in the next few years. For this reason, the clinician has the task of early diagnosing neuropathy and offering the patient the most suitable therapeutic measures to prevent or delay the progression of the complication or to treat the symptoms.

Based on the nervous system involved, autonomic or peripheral, there are various manifestations of DN, and consequently related to the type of nerve (large or small fibers) and organ involved (heart, bladder, intestine, etc.) [12]. DPN can occur as symmetric sensory-motor axonal neuropathy, proximal asymmetric painful motor neuropathy, mononeuropathy, and autonomic neuropathy, this latter mainly through the involvement of small fibers [13]. Among them and among the various forms of DN, symmetric sensory-motor axonal DPN is the one with the highest prevalence [13].

The pathophysiology is characterized by predominantly metabolic/inflammatory damage that affects the peripheral nerves responsible for conducting the motor and sensory impulse [14,15]. This microvascular complication is defined as “length-dependent” neuropathy since it affects the longest nerve fibers more frequently [16]. 

DPN is the main risk factor for the development of lower limb ulcers and amputations and subsequent disabling [2]. This event has devastating effects on the quality of life and prognosis of patients and has a relevant impact on health care costs (almost EUR 837–962 million for ulcers and amputation in 2014–2015 in England) [17]. Moreover, in a Scottish national observational study on almost 17,000 diabetic subjects, those with increased foot risk showed a lower 2-year amputation-free survival compared with subjects who had no previous ulcer (*p* < 0.0001) [2,18].

To prevent these terrible complications and to reduce the costs of the health care system, adequate prevention, and therefore an early proper diagnostic screening, seems essential [19]. Knowledge of the pathophysiological and diagnostic aspects of DPN is therefore essential to pursue adequate clinical practice. 

The purpose of this review is to shed light on the most recent discoveries in the pathophysiological and diagnostic fields concerning this complex and frequent complication of DM.

## 2. Pathophysiology of Peripheral Neuropathy in Diabetes

The pathophysiological mechanisms through which diabetes causes neuropathic damage are many and involve various metabolic and intracellular signaling processes, still today not fully understood. 

Axonal degeneration, with both primary and secondary demyelination, has been documented in nerve biopsies from both animal models and individuals with diabetic polyneuropathy [20,21]. In particular, impairment of the myelin sheath and Schwann cells has been shown. These latter dissociate from axons both in myelinated and unmyelinated neurons [22]. Consequently, axonal impulse conduction and signaling are disrupted, together with a reduction of neurotrophic factors, thus resulting in centripetal degeneration and distal axonal loss that progresses length-dependently [22]. This indicates that the longest nerve fibers (e.g., sciatic and sural nerves) are those at the greatest risk of being involved [22,23].

Among the postulated mechanisms, an inflammatory process seems to induce nerve damage through metabolic and cellular pathways. Moreover, other metabolic risk factors related to diabetic status seem to be associated. 

**Figure 1:**
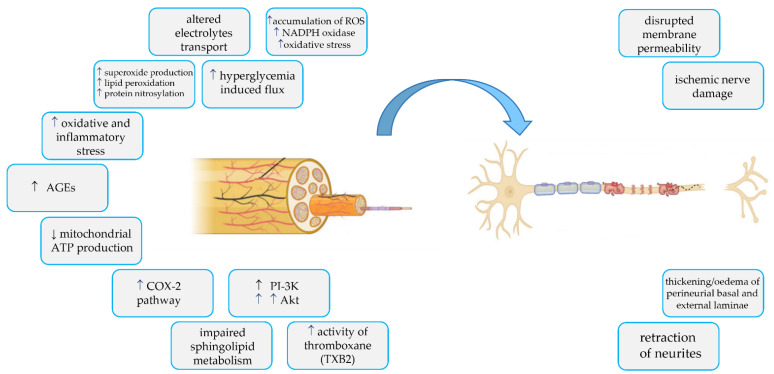
Main pathophysiological mechanisms of DPN.

Experiments on animal models and on subjects with DM could clarify some of these aspects to better understand the pathophysiology of this complication. Below, the main pathophysiological mechanisms are described, to date known as responsible for the development of DPN (Figure 1).

### 2.1. The Nerve Barrier Disruption

The microvessels of peripheral nerves are lined by a blood–nerve barrier (BNB) within the presence of the peripheral neural parenchyma (endoneurium). The components of this barrier are the endothelial cells bound by tight junction proteins, pericytes, and basal lamina [24,25]. This barrier constitutes an important structure of energy supply by expressing transporters for nutrients and constitutes a defense for the nerve [26].

Altered BNP function seems to be the first marker of damage responsible for the development and progression of DN. The increased permeability of the damaged barrier facilitates the passage of high-molecular-weight proteins, such as albumin and immunoglobulin G (IgG), into the endoneurium [27,28]. The hyperglycemia-induced flux, through the polyol pathway, promotes disrupted membrane permeability. This latter is in turn responsible for altered regulation of the protein molecules and electrolyte transport, thickening of perineurial basal or external laminae, and therefore edema [29]. The progressive deposition of edema promotes subsequent ischemic nerve damage [29]. 

Furthermore, as evidenced in sural nerve biopsies, the endothelial cells of the small endoneurium vessels undergo swelling and, together with thickening of the basal lamina, result in a reduction of the vascular lumen. The biopsies also showed a reduction of the tight junctions, an accumulation of fibrin, and a reduction of the pericytes that circumscribe the vascular endothelium. Ischemia induced by this disruption could stimulate macrophages to release vascular endothelial growth factors (VEGFs) [30,31].

### 2.2. Role of Inflammation

The systemic inflammatory status detected in people with DM appears to underlie the development of diabetic neuropathy. Cytokines, inflammatory cells, and growth factors have been proposed as mediators of the development of this complication in both animal and human models [32,33,34]. In particular, hyperglycemia appears to be associated with the stimulation of the cyclooxygenase-2 (COX-2) pathway in micro-vessels, with subsequent development of oxidative and inflammatory stress at the level of peripheral nerves [32]. The persistence of diabetes for 6 months in diabetic mouse models with active COX-2 gene resulted in increased oxidative and inflammatory stress, reduction of nerve conduction velocities, and intraepidermal fiber density, compared to mice with inactive COX-2 gene [32]. Moreover, hyperglycemia seems to be linked to the upregulation of COX-2 with an increase in the vasoconstrictive activity of thromboxane (TXB2) and prostaglandins (PGE2) concentrations, reduction of prostacyclin, increased superoxide production, and subsequent lipid peroxidation and protein nitrosylation [35,36,37,38]. In another experimental study on mice with spinal cord injury, the administration of interleukin-1beta (IL-1beta), IL-6, and tumor necrosis factor alpha (TNF alpha) determined the activation of macrophages and microglial cells, thus increasing the inflammatory response [39]. Moreover, in individuals with diabetes, some authors have found a significant association between the serum concentrations of soluble intercellular adhesion molecule (sICAM)-1 and IL-6 and painful diabetic neuropathy [40].

Other authors suggest the role of reactive oxygen species (ROS), including mitochondrial overproduction of superoxide, in the development of microvascular complications. In a study on subjects with T1DM and T2DM, low concentrations of antioxidants superoxide dismutases (SODs) and glutathione (GSH) were significantly associated with the presence of DPN (β = −0.206, *p* = 0.0003) [41].

### 2.3. Role of Advanced Glycation End Products (AGE)

In addition, a relevant role in the development of neuronal damage seems to be determined by the increased amount of AGEs, which are highly expressed in people with hyperglycemic status. AGEs are generated through the nonenzymatic aggregation of glucose, or other saccharides, to proteins, lipids, and nucleotides and tend to accumulate in the perineurium, endothelial cells, pericytes of endoneurial micro vessels, and myelinated and unmyelinated fibers [42,43,44].

Some in vitro experimental studies show how the exposure of neurons, particularly of the dorsal root ganglia, to AGEs can determine an increase in their receptors (RAGE) on the neuronal surface [45]. The subsequent activation of the receptors due to binding with their ligand would result in downstream signaling through the mitogen-activated protein kinase (MAPK) pathway and the phosphoinositide-3 kinase (PI-3K) pathway, which results in the phosphorylation and activation of Akt [45]. The activation of these intracellular pathways determines the accumulation of ROS, the activation of NADPH oxidase, and oxidative stress. Such mechanisms induce neuronal damage and apoptosis, thus explaining the role of AGEs in the development of diabetic neuropathy, as well as retinopathy and diabetic nephropathy in other tissues [46,47].

### 2.4. Role of Lipids

Some recent studies have focused on the association between metabolic syndrome, often present in subjects with T2DM, and the development of DPN [48]. In particular, this association seems mainly associated with small unmyelinated axon damage. However, in a small proportion of individuals, large fibers could also be affected [48]. 

As described above, a fundamental role in the development of microvascular complications of diabetes is played by oxidative stress and the production of ROS, deriving, in the first instance, from hyperglycemia status [49]. Plasma lipids thus become targets of ROS, a process that in turn tends to fuel the peroxidation of the lipids themselves. The end products of this peroxidation process, lipid peroxides, can be toxic at the cellular level, including neurons [49].

Recently, some authors have investigated the presence of potential biomarkers of DPN and, in particular, have evaluated whether there are any metabolic syndrome-derived metabolites associated with DPN. The results suggest that in individuals with DPN, some biomarkers (e.g., perfluorooctanoate, sulfate of piperine metabolite, isoursodeoxycholate sulfate, N-acetyl-3-methylhistidine, tartarate, citrate, N-acetyl-beta-alanine, ximenoylcarnitine) are associated with alterations in plasma lipid metabolites [50]. In particular, T2DM DPN plasma analysis revealed a significant reduction in complex lipids, mainly acylcarnitine and sphingolipid species, compared to T2DM subjects without polyneuropathy. Thus, it seems that the disorder of DPN metabolism could be linked to sphingolipid metabolism [50,51]. Lipid metabolites could affect mitochondrial function within nerves through a bioenergetics overload, an impairment of the citric acid cycle, and a consequent reduction in both acylcarnitine and citrate levels, with a reduction in mitochondrial ATP production [50,51]. In addition, in preclinical models of nerve regeneration, Sphingosine-1-Phosphate, through the activation of its receptor, seems linked to the phosphorylation of collapsin response-mediated protein-2, which in turn induces the rapid retraction of neurites [52]. 

### 2.5. Other Mechanisms 

Recently, other mechanisms have been hypothesized to explain the etiology of DPN and, particularly, an autoimmune etiology, and the role of genes have been suggested. 

Some authors have observed reduced peripheral nerve function and a higher risk of developing DPN in subjects with T1DM and high GAD65 antibodies, thus suggesting a common mechanism for β-cell and neuronal damage [53]. 

In a study by Janahi et al. that enrolled a mixed population with both T1DM and T2DM subjects, those with DPN had a significantly higher share of positive antinuclear antibodies (ANA) [54]. Autoimmunity could therefore have a role in determining the destruction of neurons by the same autoimmune process in T1DM, thus triggering or worsening DPN. However, other studies have not confirmed this hypothesis and have not shown any significant association between autoimmunity and the development of DPN [55,56]. 

Genetic research in this field mainly aims to find genetic alterations, variants, or biomarkers associated with an increased risk of developing DPN, to give insights into the pathogenesis, and to find responders to treatment [57]. Moreover, researchers have paid attention to the possible role of miRNAs, small gene regulators, in the development of diabetes and its micro- and macrovascular complications [58]. For what concerns genes, a role of such as angiotensin converting enzyme (ACE) gene, methylenetetrahydrofolate reductase (MTHFR) gene, glutathione peroxidase-1 (GPx-1) gene, catalase (CAT) gene, and genes associated with the pentose phosphate pathway has been proposed [57]. Human studies have explored the association between miRNAs and the development of DPN. In particular, miRNAs related to mechanisms of inflammation, insulin signaling pathways, adipogenesis, and lipolysis have been explored. In the plasma of subjects with DPN, miRNAs (e.g., miRNA-199a-3p, miRNA-128a) are upregulated, while others (e.g., miRNA-155, miRNA-499a) are downregulated [59,60]. 

However, further studies seem necessary, and up to now, no clear association has been found between genes and miRNA alterations and the risk or pathogenesis of DPN.

## 3. Diagnosis 

The diagnosis of DPN is mainly clinical, and a screening visit should be performed at least once a year in T1DM 5–10 years after diagnosis, while even from the time of diagnosis in T2DM subjects [61]. The history of the patient, together with screening scores and neurological physical examination of the lower limbs for the evaluation of symptoms and signs, could exclude other types/causes (e.g., alcoholic, vasculitic, nutritional and drug-induced/toxic neuropathies, tarsal tunnel) of peripheral neuropathies and identify the typical characteristics of the DPN [61]. In more complex cases, when typical symptomatology/signs are missing, or when many other confounding comorbidities are present, nerve conduction studies (NCS) and skin biopsies, respectively, to identify large or small fiber neuropathy, could be necessary [13]. According to the literature, the presence of symptoms or signs of neuropathy defines the picture of possible DPN. It is probable that signs and symptoms are present together, confirmed if symptoms and/or signs are associated with alterations in nerve conduction studies [62]. In the case of a small fiber impairment, nerve conduction studies are normal, usually together with the absence of neurological signs. In these cases, sweat testing, quantitative sensory testing (QST), and a skin biopsy can be performed to detect small fiber involvement. The choice between these techniques could depend on the availability of expertise at clinical centers [63]. The main fibers and diagnostic tools involved are summarized in Table 1.

### 3.1. Screening Scores

Currently, various validated scores are used for the evaluation of symptoms and signs of DPN, and, according to the diagnostic criteria, to identify the forms a possible or probable [2,62]. The scores described below mainly assess tactile and vibratory sensitivity and deep reflexes, thus describing the clinical assessment of the integrity of the large nerve fibers (myelinated alpha and beta). However, as described in the last part of this section, some recent scores could help clinicians evaluate small fibers.

Dolor neuropatique 4 (DN4) is one of the most widely used validated scores to assess the main symptoms and signs of neuropathic pain and painful diabetic neuropathy [64]. According to some authors, it allows the identification of neuropathic pain with a sensitivity of 80% and a specificity of 91% through the evaluation of 10 items and with a cut-off of 4 (burning, feeling cold, tingling, sensation of electric shock, pinprick, numbness, itching, hypoesthesia to the touch, puncture, touch of painful skin) [65,66]. For these reasons and based on the results and experience gained by clinicians over the years, DN4 is still suggested as useful by the guidelines, particularly for the screening of neuropathic pain, even in subjects without DM [67]. 

The Michigan Neuropathy Screening Instrument (MNSI) is a simple and non-invasive test that allows one to perform a neurological examination. This score aims to highlight the specific signs of DPN (tactile sensitivity, vibration, osteotendinous reflexes, dry skin, presence of ulcers) [13,68]. In addition to an accurate inspection of the foot, the score allows the detection of alterations of deep sensitivity through the 128 Hz tuning fork, of the Achilles tendon reflexes, and of tactile sensitivity through the 10 g monofilament. Moreover, the other items aimed to evaluate the presence of typical signs of neuropathy (feet numb, burning pain, muscle cramps, etc.) [68,69]. Some authors have shown that, with a cut-off of 4, the sensitivity of the Michigan Neuropathy Screening Instrument is 40%, while the specificity is 92% [68]. 

The Toronto Clinical Neuropathy Score and the modified Toronto Clinical Neuropathy Score (mTCNS) are simple and non-invasive and have been used in some trials. These are still indicated and validated for screening diabetic neuropathy and other clinical manifestations of neuropathic pain [2,70,71]. Foot pain, numbness, tingling, weakness, and ataxia are the main evaluated symptoms, while the evaluated signs are sensitivity to pinprick, temperature, light touch, vibration, and position sense. This score showed good reliability, validity, and inter-rater reliability (95% confidence interval, 0.79–0.91) [72]. Furthermore, this score has recently been used to evaluate the efficacy of drugs in reducing symptoms of DN [73].

The Neuropathy Disability Score is still one of the scores validated for the screening of neuropathy and has been used in some trials [74,75,76]. This score evaluates peripheral sensitivity and tendon reflexes and is still indicated among those useful for the screening for neuropathy [77]. 

Regarding the evaluation of small fiber neuropathy (SFN) (small myelinated Aδ-fibers and unmyelinated C-fibers), recent literature suggests the Small Fiber Neuropathy and Symptoms Inventory Questionnaire. This latter includes 13 items: changed sweating pattern, constipation, diarrhea, micturition problems, dry mouth, dizziness on standing from sitting or supine position, dry eyes, palpitations, hot flashes, sensitive skin, heat intolerance, burning feet, and restless legs. For each item, four response options are present: 0 = never, 1 = sometimes, 2 = often, and 3 = always. This score thus allows for an assessment of the state of integrity and symptoms of SFN, regardless of the cause [63,78]. 

### 3.2. Quantitative Sensory Testing and Neurological Tools 

Quantitative sensory testing (QST) is a useful tool in clinical practice, complementary to physical examination and scores, and it is capable of identifying signs of altered sensitivity in individuals with suspected neuropathy. In particular, QST can be performed to evaluate the functional impairment of sensory nerve fibers (Aδ-fibers, Aβ-fibers, and C-fibers) [79,80]. QST is a non-invasive method that can be used to assess the features of neuropathic pain and to evaluate both loss and gain of sensory function [63].

In clinical practice, QST could be used to screen large and small fiber neuropathies, monitor somatosensory deficits, and of evoked pains, allodynia, and hyperalgesia [81]. However, QST alone does not allow us to reach a diagnosis of neuropathy and neuropathic pain [82]. 

Vibratory sensitivity is lost early in the onset and progression of DN, and for this reason, confirmation of its reduction allows the identification of the complication. In addition to the use of the 128 Hz tuning fork used to complete the screening scores, some neurothesiometers and biothesiometers are still used [83]. The 128 Hz tuning fork alone has limitations, such as the presence of inter- and intra-individual variability (ranging from none to strong; κ = 0–0.86) [84,85,86]. 

In neurothesiometer and biothesiometer testing, the arm of the device with vibratory stimulus is applied to the apex of the hallux, and the amplitude of vibration gradually increases until the subject perceives the vibration [87]. However, also for what concerns neurothesiometers and biothesiometers, some studies have reported variable reliability (κ = 0.58–0.65; κ = 0.51–0.61) [86,88]. Furthermore, each test can be affected by the operator’s ability and by the patient’s psychological factors [89]. Moreover, subjects with severe neuropathy are often encountered because the maximum vibration amplitude that can be produced cannot be detected. For clinicians, it is thus hard to distinguish the vibration sensitivity threshold among those subjects [88]. Other authors reported that neurological alterations highlighted by neurothesiometer are often more severe in individuals with other renal and ocular microvascular complications (*p* < 0.001). Additionally, for these reasons, the authors suggested that this tool is still precious in monitoring the DPN status and the prognostic evaluation of the subject [90].

VibraTip is another tool that allows for the evaluation of vibration sensitivity. This portable device is applied to the pulp of the hallux and on the first and third metatarsal heads and provides a stimulus of 128 Hz [91]. It showed good agreement with the Neuropathy Disability Score (NDS) and the Ipswich Touch Test (IpTT) (*p* < 0.001) [92]. Moreover, VibraTip was compared against a biothesiometer and provided a positive predictive value of 90.3%, specificity of 84.2%, and sensitivity of 50%. When VibraTip and 10 g of monofilament were combined, the sensitivity improved to 62.5% [93].

Similar results were also found in another study in which VibraTip was compared with IpTT and the neurothesiometer. Compared with the latter, it demonstrated good agreement (κ = 0.713, *p* < 0.001) [94].

The International Working Group on the Diabetic Foot (IWGDF) guidelines recommend using IpTT if a 10 g monofilament is unavailable [95]. IpTT could be useful for detecting any loss of protective sensation in the feet of subjects with DM [96]. Sensitivity assessment points are placed at the tips of the first, third, and fifth toes of both feet, and the other two sites are the dorsum of both halluces. IpTT, compared to the 10 g monofilament and the vibration perception test, has shown good sensitivity and specificity, ranging from 51 to 100% and 90 to 98%, respectively [97]. 

The Neuropad, an adhesive pad that contains blue salt anhydrous cobalt II chloride, is a tool that allows the evaluation of sweat dysfunction of small fibers. As mentioned above, small fibers represent 70% of the innervation; thus, describing their impairment is important to diagnose DPN early [98,99]. C fibers are responsible for thermal transmission, pain, and the autonomous function of sweating. Based on this neurophysiological principle, the Neuropad, through an evaluation of the sweating of the skin of the sole of the foot, allows for an inexpensive evaluation of the function of small fibers and signs of neuropathy [100,101]. An indicator test is applied to a callus-free area on the plantar surface of the feet at the level of the first to the second metatarsal heads. After 10 min, an evaluation of the indicator is done to verify the color change [102]. As described by some authors, compared with the Diabetic Neuropathy Index (DNI), the indicator test showed sensitivity of 94.4% and specificity of 69.7% for diagnosing peripheral neuropathy [102]. Moreover, compared with 10 g monofilament and 128 Hz tuning fork, the Neuropad has shown a sensitivity of 94% and specificity of 29% [103]. According to these results, in another recent study enrolling a diabetic population, Neuropad showed good sensitivity, accuracy, and specificity in detecting DPN compared to the Michigan Neuropathy Screening Instrument Questionnaire and biothesiometer. In the same study, Neuropad showed high sensitivity but moderate specificity compared with 10 g monofilament [104].

The Sudoscan is a tool that allows for a quantitative evaluation of cutaneous sudomotor function through reverse iontophoresis and chronoamperometry [105,106,107,108,109]. During the examination, the subjects placed their palms and soles on a platform for a few minutes. The platform is connected to low-voltage electrodes (<4 V), which stimulate the activity of sweat glands [110,111]. The subsequent production of chloride ions generates an electrochemical reaction, which is measured as electrochemical skin conductance in micro-Siemens (uS) [112]. In this manner, a quantitative measurement of the activity of the sweat glands and, therefore, of sympathetic cholinergic and thin unmyelinated C-fibers of the autonomic nervous system is carried out [112]. Recently, some authors have analyzed data from a population of 144 subjects with T2DM by comparing the discrimination power of various instruments for detecting DPN. At the end of the study, the Sudoscan-only model showed good discrimination power compared to MNSI and monofilament 10 g [113]. Moreover, the Sudoscan plus MNSI model showed higher areas under the receiver operating characteristic curve (AUC) than the MNSI-only model (0.717 vs. 0.638, *p* = 0.011) [113]. This result demonstrates that since each score evaluates more particularly different fibers, large and small fibers, their association can increase the diagnostic accuracy in detecting DPN. A recent cross-sectional study included 49 healthy subjects (group I), 75 hypertensive individuals without T2DM (group II) and 76 hypertensive subjects with T2DM (group III). Sudoscan revealed that the electric skin conductance values of the peripheral sudomotor nerves were significantly lower in groups II and III than the corresponding values of Group I. In addition, the authors demonstrated an association between the presence of peripheral nerve impairment detected by Sudoscan and the risk of developing hypertension and cardiac autonomic neuropathy. For this reason, the authors hypothesized that the capability of Sudoscan in detecting the impairment of peripheral autonomic fibers could also be useful as a prognostic indicator of cardiac autonomic damage [114].

The Nervecheck Master is another portable device useful as a QST for evaluating DPN. In particular, this tool allows an evaluation of vibration sensitivity, pain sensitivity, and thermal sensitivity. The instrument is equipped with two mechanical stimulation arms, one for vibration sensitivity and the other for pain and thermal (cold/warm) stimulation [115]. The tool has been compared with the Neuropathy disability score (NDS), NCS, and the intraepidermal and corneal nerve fiber density (IENFD and CNFD) methods. The Nervecheck score for the vibration perception threshold has shown good diagnostic accuracy with reference to NCS (area under the curve [AUC]: 82–84%) and moderate with reference to IENFD and CNFD. The diagnostic accuracy of cold/warm perception threshold was moderate with reference to NCS, IENFD, and CNFD (AUC: 69–78%). Low accuracy has been observed for neuropathic pain (AUC: 63–65%) [116]. Recently, the Nervecheck Master has also been used to diagnose other forms of neuropathy, but the results seem conflicting and require further investigation [117,118].

### 3.3. Nerve Conduction Studies (NCS)

Nerve conduction studies (NCS) are the gold standard exam for diagnosing DPN [119]. NCS allows an evaluation of the large fibers (myelinated alpha and beta), while with this tool it is not possible to identify the function of the small fibers (small myelinated Aδ-fibers and unmyelinated C-fibers) [120,121]. The application of skin electrodes permits nerve stimulation in μV and the detection of electrical nerve activity through a series of parameters that are useful for diagnosing nerve and muscle fiber pathologies [120,122,123,124,125]. The parameters mainly used are velocity nerve conduction, amplitudes, and latencies. Particularly in diabetic neuropathy, sensory nerve action potentials (SNAP) detect the activity of sensory nerve fibers from the distal receptors in the skin to the dorsal root ganglia. The compound muscle action potentials (CMAP) describe motor nerve fiber activity, from the anterior horn cell to the termination along muscle fibers [126]. The description of these conduction characteristics allows us to differentiate the underlying nerve pathophysiology as either axon loss or demyelinating impairment [126]. For the evaluation of DPN, the nerve conduction study of the sural nerve has been shown to be the most sensitive, and some authors have also suggested that the study could also be useful as a measure of DPN screening, severity evaluation, and follow-up [127,128]. This is related to the anatomical characteristics of the nerve and to the pathophysiological modalities of the progression of nerve damage in DPN. As mentioned above, DN is recognized as a length-dependent process, and thus the earliest abnormalities appear at the extremities of the lower limbs, where the ends of the longest nerve fibers (plantar nerves, peroneal nerves, sural nerves) are represented [16]. In particular, many authors agree that the earliest impairment of DN involves the sural nerve [129]. Furthermore, the sural nerve, being a nerve with a large diameter, allows for the acquisition of greater amplitudes and conduction velocities, which can therefore allow for better discernment between the various degrees of damage in subjects with DM compared with healthy controls (SNAP amplitude (mV) 8.0 ± 3.6 vs. 11.1 ± 4.7; *p* < 0.0001) [129].

Some authors suggest that the study of fibers even more distal than the sural, such as the fibers of the plantar nerve, could be an early detector of diabetic neuropathy [129,130,131,132]. Others highlight how the joint study of peripheral nerves together, medial plantar and peroneal, in addition to dorsal sural NCS, could increase sensitivity in the detection of neuropathy, thus allowing earlier diagnosis, especially when routine NCS are normal [129,133].

However, compared to the conventional study of other nerves, the sural nerve, due to its anatomical position, is less subject to mechanical trauma and therefore allows a technically easier and more sensitive procedure [134]. Furthermore, conventional surface recording NCS of the dorsal sural nerve has demonstrated the same diagnostic power as the sural near-nerve needle technique (NNT), a technique that allows direct evaluation of the nerve through a fine needle electrode. In the same study, sural NCS with both NNT and surface recording demonstrated good specificities (85–95%) and positive predictive values (94–98%), while specificity for the medial plantar nerve was lower (68%) [135].

### 3.4. Corneal Confocal Microscopy, Skin Biopsy, Laser-Evoked Potentials

As mentioned, a series of complementary tests are required for the evaluation of small fibers, which allows clinicians to expand their diagnostic capabilities.

In recent years, corneal confocal microscopy (CCM) has been suggested as a useful tool to screen, diagnose DPN, and for patient follow-up [136,137]. This is a non-invasive ophthalmic application capable of measuring the branch density and length of small corneal nerve fibers [2]. Some authors have observed that in subjects with DPN, CCM measures worsen early and with greater magnitude than large fibers, autonomic and IENFD measures of neuropathy. Thus, CCM would be useful for reaching an early diagnosis of DPN before the development of large fiber and autonomic symptom/signs of DPN [138]. Moreover, in some studies, CCM impairment has been associated not only with the worsening of other small fiber measures (cold perception threshold, IENFD, and autonomic neuropathy), but also with large nerve impairment, evaluated through nerve conduction studies (NCS) [138,139,140,141]. Furthermore, in validation studies of people with DM, CCM has shown variable sensitivity and specificity results (almost 82% and 70%, respectively). For this reason, CCM is still not considered a point-of-care device and is used in specialist units [2,142,143]. A recent study on 261 subjects with T1DM and T2DM evaluated the risk of developing DPN in those with normal electrophysiology and corneal confocal microscopy at baseline. After a mean follow-up time of 5.8 years, DPN developed in 60 participants (23%; 4.29 events per 100 person-years) and was associated with T2DM, higher BMI, longer duration of diabetes, and lower baseline NCS and CCM parameters. The authors have observed that corneal nerve fiber length of 14.1 mm/mm^2^ results as the optimal diagnostic threshold, associated with 67% sensitivity, 71% specificity, and a hazard ratio of 2.95 (95% CI 1.70–5.11; *p* < 0.001) for new-onset DPN. These values seem coherent with previous results obtained from other studies [144]. However, not all authors agree in observing this association; thus, further studies seem necessary to better confirm the results. Moreover, this tool is currently available only in a few selected units. This lack of diffusion severely limits the accessibility of people to this diagnostic tool and the possibility of briefly expanding knowledge in this field.

Punch skin biopsy is necessary to observe IENFD, which, together with two clinical signs and abnormal QST, represents the gold standard for diagnosing SFN [63,145]. Its use is also extremely useful when symptoms are few or not typical and QST are normal or borderline [63,145]. According to guidelines, punch biopsy is performed acquiring 3 mm of skin 10 cm above the lateral malleolus, at the level of the sural nerve surface of innervation. Thereafter, bright-field immunohistochemistry and quantification of linear IENFD in three 50-µm sections is performed [146,147,148]. Despite the importance of this diagnostic process, skin biopsies are available in a few selected centers and with specialized personnel [63]. Likewise, the CCM, the skin biopsy, permits observation of the IENF and has been proposed for a diagnostic evaluation and monitoring of SFN. However, due to the lack of specialized clinicians in performing this technique, this latter role remains to be discussed or not really feasible. In people with diabetes, early IENFD loss has been observed, and it is considered a main sign of SFN [148,149]. However, not all authors agree that the severity of IENFD loss correlates with an increase in sensory thresholds and the severity of the disease [150,151]. In a recent detailed skin biopsy analysis, the aim of the study was to evaluate the association between skin biopsy findings, pain intensity, and QST. At the end of the study, an association was observed between pain and increased density of dermal peptidergic fibers containing substance P, but there was no difference in intraepidermal nerve fiber density and quantitative sensory testing results between subjects with and without pain [152]. A recent analysis involving 245 subjects with SFN compared different diagnostic methods, including skin biopsies. This latter showed moderate sensitivity (58%) and excellent specificity (91%). Moreover, the diagnostic accuracy increased to a sensitivity of 90% and a specificity of 87% when skin biopsy was associated with the other diagnostic tools (QST, Electrochemical Skin Conductance, Laser Evoked Potentials). Therefore, as described elsewhere, the authors pointed out the importance of combining various methods to reach the diagnosis of SFN [153].

Skin biopsy has also been used as the gold standard for evaluating the diagnostic accuracy of laser evoked potentials (LEPs), a new noninvasive neurophysiological method for evaluating SFN and nociceptive pathways (A delta fibers) [154]. Particularly, this is described as a noninvasive tool that generates a highly collimated infrared beam (wavelength 10.6 lm) on a surface area ranging 0.8–20 mm^2^, with a duration of 10–50 ms and an energy density of 10 mJ/mm^2^. Brain-evoked potentials were recorded from 19 Ag-AgCl scalp electrodes, with linked earlobes (A1A2) as the reference and on a PL-EEG [154,155]. In a population of diabetic subjects and compared punch skin biopsy, LEPs performed had sensitivity (91%), specificity (83%), and area-under-the ROC curve (0.924). In another study, which enrolled 100 subjects with DN, also here compared with skin biopsy, LEPs shown 78% sensitivity and 81% specificity in the diagnosis of diabetic SFN [156]. Thus, the authors concluded that LEPs could represent a valid and less invasive alternative to punch skin biopsy for diagnosing SFN [156].

## 4. Conclusions

DPN is one of the main microvascular complications of diabetes mellitus. Although pathophysiological processes may differ between T1DM and T2DM, it is currently difficult to highlight significant differences. The pathophysiology underlying the development of this complication involves various cells and tissues in a mix of pathways that together seem to lead to its development [1]. The diagnostic procedure and the main most up-to-date treatment modalities are well described by the last compendia from the American Diabetes Association [1]. Despite considerable progress in discovering these pathways, further studies seem necessary to better clarify the development of this complication, especially regarding the role of endothelial dysfunction and the inflammatory cascade [157,158].

Regarding the diagnosis of DN, in most cases, thanks to clinical evaluation and scores and to the use of some non-invasive tools (e.g., QST), it seems possible to reach a diagnosis of DPN, both for the involvement of large and small fibers. However, when the symptoms/signs are more nuanced and when other comorbidities are present, NCS, for the involvement of the large fibers, and punch skin biopsy, for the involvement of small fibers, seem necessary to reach the diagnosis of DPN.

Moreover, further studies are necessary to find specific and effective therapies for DN, as developed for other complications [159,160,161,162].

## Figures and Tables

**Table 1:** Dolor neuropatique 4; MNSI, Michigan Neuropathy Screening Instrument; mTCNS, modified Toronto Clinical Neuropathy Score; NDS, Neuropathy Disability Score; SFN-SIQ, Small Fiber Neuropathy and Symptoms Inventory; Questionnaire; IpTT, Ipswich touch test; NCM, Nervecheck Master; NCS, Nerve Conduction Studies; CCM, Corneal Confocal Microscopy; IENFD, intraepidermal nerve fibers density; LEPs, laser evoked potentials.

Type of Fibers	Stimulus	Screening Scores	Quantitative Sensory Testing and Neurological Tools	Nerve Conduction Studies/Others	Sensitivity/Specificity
**Large** **(sensory axons)** **- myelinated α** **- myelinated β**	Tactile Vibration Position	DN4, MNSI, mTCNS, NDS MNSI, mTCNS MNSI, NDS	10 g monofilament, IpTT Neurothesiometer/biothesiometer VibraTip	NCS NCS	DN4 (80/91%) MNSI (40/92%) 10 g monofilament (62.5%) IpTT (50–100/90–98%) Neurothesiometer/biothesio meter (reliability; κ = 0.58–0.65; k = 0.51–0.61) VibraTip (50–62.5/84.2%) NCS (85/95%)
**Small** **(sensory axons)** **- Aδ-fibers** **- Unmyelinated** **C-fibers**	Tactile Cold Pressure Warm thermal Pain Sweating	SFN-SIQ SFN-SIQ DN4, mTCNS, SFN-SIQ MNSI, mTCNS, SFN-SIQ	NCM NCM NCM Neuropad, Sudoscan	CCM, IENFD, LEPs CCM, IENFD, LEPs	Neuropad (~90/~70%) Sudoscan (~90%) NCM (AUC 60–85%) CCM (67/71%) IENFD (90/87%) LEPs (91/83%)

## Data Availability

Not applicable.

## Abbreviations

Advanced glycation end products receptors (RAGE); advanced glycation end products (AGE); blood-nerve barrier (BNB); cyclooxygenase-2 (COX-2); compound muscle action potentials (CMAP); corneal nerve fiber density (CNFD); diabetes mellitus (DM); diabetic neuropathy (DN); diabetic peripheral neuropathy (DPN); dolor neuropatique 4 (DN4); immunoglobulin G (IgG); interleukin-1beta (IL-1beta); intraepidermal nerve fiber density (IENFD); glutathione (GSH); mitogen-activated protein kinase (MAPK); Michigan Neuropathy Screening Instrument (MNSI); nerve conduction studies (NCS); peripheral neuropathy (PN); phosphoinositide-3 kinase (PI-3K); prostaglandins (PGE2); quantitative sensory testing (QST); reactive oxygen species (ROS); soluble intercellular adhesion molecule (sICAM)-1; small fiber neuropathy (SFN); sensory nerve action potentials (SNAP); superoxide dismutases (SODs); thromboxane (TXB2); tumor necrosis factor alpha (TNF alpha); type 1 diabetes mellitus (T1DM); type 2 diabetes mellitus (T2DM); vascular endothelial growth factors (VEGFs).
